# Transposable element evolution in *Heliconius* suggests genome diversity within Lepidoptera

**DOI:** 10.1186/1759-8753-4-21

**Published:** 2013-10-02

**Authors:** Christine A Lavoie, Roy N Platt, Peter A Novick, Brian A Counterman, David A Ray

**Affiliations:** 1 Department of Biochemistry, Molecular Biology, Entomology and Plant Pathology, Mississippi State, MS 39762, USA; 2 Institute for Genomics, Biocomputing and Biotechnology, Mississippi, MS 39762, USA; 3 Department of Biological Sciences and Geology, Queensborough Community College, Bayside, New York, NY 11364, USA; 4 Department of Biological Sciences, Mississippi State, MS 39762, USA; 5 Current Address: Department of Biological Sciences, Texas Tech University, Lubbock, TX 79409, USA

**Keywords:** *Heliconius melpomene*, Lepidopteran, Butterfly, Transposable elements, Genomic deletions

## Abstract

**Background:**

Transposable elements (TEs) have the potential to impact genome structure, function and evolution in profound ways. In order to understand the contribution of transposable elements (TEs) to *Heliconius melpomene*, we queried the *H. melpomene* draft sequence to identify repetitive sequences.

**Results:**

We determined that TEs comprise ~25% of the genome. The predominant class of TEs (~12% of the genome) was the non-long terminal repeat (non-LTR) retrotransposons, including a novel SINE family. However, this was only slightly higher than content derived from DNA transposons, which are diverse, with several families having mobilized in the recent past. Compared to the only other well-studied lepidopteran genome, *Bombyx mori*, *H. melpomene* exhibits a higher DNA transposon content and a distinct repertoire of retrotransposons. We also found that *H. melpomene* exhibits a high rate of TE turnover with few older elements accumulating in the genome.

**Conclusions:**

Our analysis represents the first complete, de novo characterization of TE content in a butterfly genome and suggests that, while TEs are able to invade and multiply, TEs have an overall deleterious effect and/or that maintaining a small genome is advantageous. Our results also hint that analysis of additional lepidopteran genomes will reveal substantial TE diversity within the group.

## Background

Transposable elements (TEs) are segments of DNA that can mobilize in a genome. They impact the structure and function of the genomes they occupy. TEs can be divided into two classes. Class I TEs are the retrotransposons, which require an RNA intermediate and use a “copy and paste” mechanism to insert themselves into a new location in the genome. Retrotransposons are further divided into two groups, the long terminal repeat elements (LTRs) and non-LTR elements. LTR retrotransposons, such as members of the Gypsy and Copia superfamilies, are similar in structure to some retroviruses. Non-LTR retrotransposons lack LTR sequences and autonomous versions (Long INterspersed Elements or LINEs) usually harbor one or two open reading frames (ORFs) that are responsible for their mobilization. Examples include the LINE1, CR1, and RTE superfamilies and can be categorized into 28 monophyletic clades [1]. Short INterspersed Elements (SINEs) are a group of nonautonomous non-LTR retrotransposons that are mobilized via the enzymatic machinery of LINEs [2].

Class II elements include the DNA transposons which use a “cut and paste” mechanism to mobilize in the genomes they occupy. Typically, DNA transposons require a transposase enzyme to recognize the terminal inverted repeats (TIRs) of the transposon and then excise and reinsert the element into another location in the genome [3]. Examples of Class II elements include members of the TcMariner, hAT, and piggyBac superfamilies. There is a second group of Class II TEs known as the rolling circle transposable elements that includes the Helitrons [4].

The first lepidopteran to have its whole genome sequenced, the silkworm moth *Bombyx mori*, has accumulated a diverse array of retrotransposons and DNA transposons [5]. For instance, a non-LTR retrotransposon, L1Bm, is abundant in the genome with copies of the 3′ end numbering ~25,000. However, like many LINEs most copies are 5′ truncated [6]. Multiple copies of a piggyBac-like DNA transposon that may harbor an intact transposase have also been found in *B. mori* and it appears to have been recently active along with other Class II elements [7].

Recently, the genome of *Heliconius melpomene* was released [8], providing new insights into lepidopteran genome evolution from a transposable element perspective. *H. melpomene* is a heliconiine butterfly that is widespread throughout Central America and South America [8,9]. The *H. melpomene* genome is the third lepidopteran and second butterfly genome to be sequenced. Unfortunately, the analysis of the second genome (and the first butterfly), the monarch, *Danaus plexipus*, was not comprehensive [10]. Therefore, we confine our comparisons of the *H. melpomene* genome to *B. mori*.

Our analyses indicate that *H. melpomene* exhibits a high rate of TE turnover, with little accumulation of older elements, especially longer, autonomous elements, suggesting that TEs have an overall deleterious effect on the genome. Furthermore, the TE landscape of *H. melpomene* is distinct compared to the silkworm moth, consisting of substantially higher Class II content and a distinct set of retrotransposons. This suggests that lepidopterans in general will exhibit high levels of TE diversity as additional genomes are sequenced and characterized.

## Results

TEs comprise ~25% of the *H. melpomene* genome (Table 1). The majority are non-LTR retrotransposons (12.07% of the genome), and among these, Short INterspersed Elements (SINEs) make up the greatest proportion (8.22%). The second most common group in *H. melpomene* are the DNA transposons, comprising 10.05% of the genome and dominated by Helitrons (~5.37% of the genome). LTR elements were also found, but occupy a much smaller proportion of the genome (0.45%).

**Table 1:** **Summary of overall transposable element content in *Heliconius melpomene***

**Class**	**Family**	**% Genome**
**DNA Transposons**		**10.05%**
	Helitron	5.37%
	Mariner	2.13%
	Tc3	1.49%
	PiggyBac	0.32%
	hobo/Activator/Tam	0.38%
	Other/Unidentified	0.36%
**LTR elements**		**0.45%**
	Gypsy	0.21%
	Copia	0.00%
	Unknown	0.24%
**Non-LTR elements**		**12.07%**
SINE	Metulj	8.22%
LINEs		3.85%
	Daphne	0.45%
	RTE	0.89%
	Jockey	0.34%
	L2	0.41%
	Zenon	0.32%
	Other/Unidentified	1.44%
**Unclassified**		**2.37%**
**Total**		**24.94%**

### Identification of Metulj and its subfamilies

**Figure 1:**
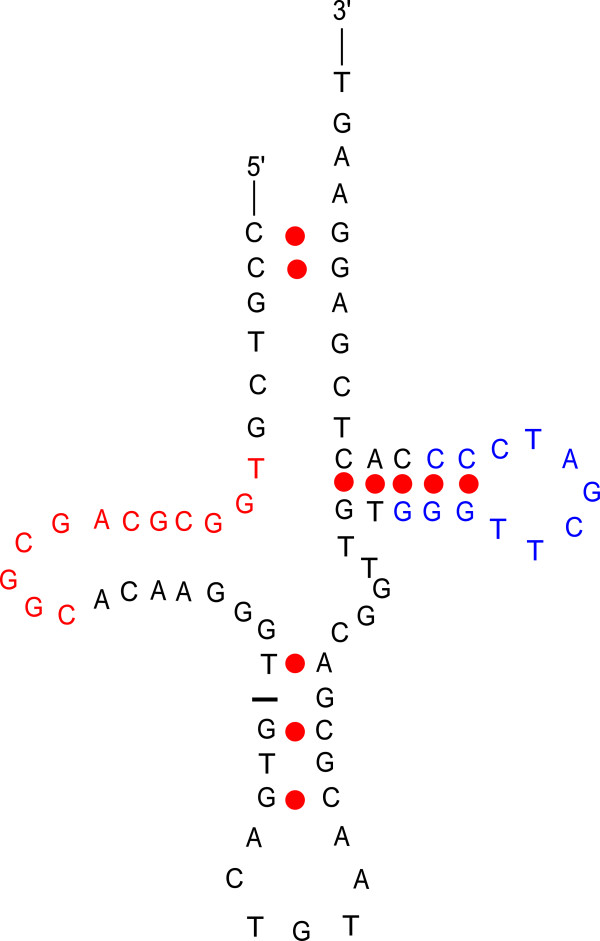
**The first 73 bases of the *H. melpomene *SINE, *Metulj*, illustrating the predicted secondary structure of the presumed tRNA-derived region.** The colored nucleotides identify the putative A (red) and B (blue) boxes typical of polymerase III promoters.

**Figure 2:**
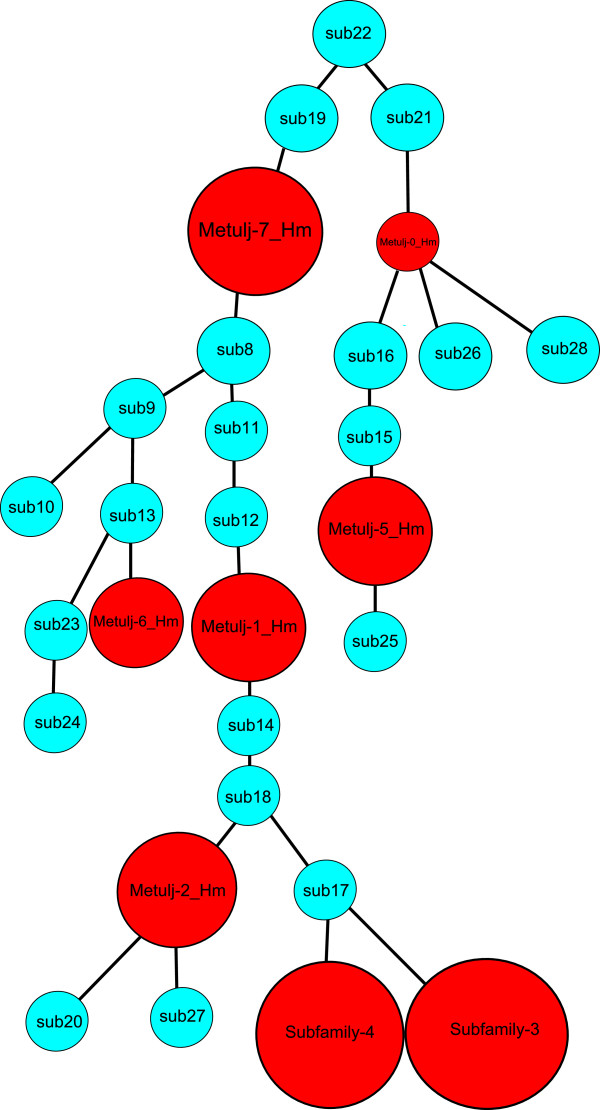
**Results of the COSEG analysis.** Red circles are proposed subfamilies.

One novel element from the genome was a SINE family we have dubbed *Metulj* (meh-TOOL), Slovenian for butterfly. The *Metulj* general consensus is ~267 bases in length with minor length differences depending on subfamily. The 5′ region of *Metulj* contains the typical RNA polymerase III promoters separated by 30 bp (Figure 1). We identified a secondary structure reminiscent of a tRNA using the methods described in [11], suggesting that the family, like many SINEs, is tRNA derived and consists of two regions, a tRNA head and a non-tRNA tail. Results from COSEG [12,13] suggest that *Metulj* comprises eight major subfamilies (Figure 2). However, subfamilies 3 and 4, appear to be composite TEs, instances where *Metulj* elements inserted into other active elements which then continued to mobilize. For example, *Metulj* subfamily 3 is embedded within a non-autonomous Mariner element, nMar-16_Hm (7,770 copies), while an unidentified repetitive sequence (21,461 copies) includes both *Metulj* subfamily 4 and a Helitron-like element (data not shown). Because these two predicted subfamilies were likely distributed throughout the genome by mechanisms other than retrotransposition, they were not included in analyses of SINE dynamics. *Metulj* subfamily 3 likely expanded as a consequence of nMar-16_Hm mobilization. Given that the identity of the repetitive element into which *Metulj* subfamily 4 has embedded is unknown, we cannot speculate on its expansion mechanism.

### Age analyses and relative insertion rates

Divergence estimates indicate that the majority of *Metulj* activity occurred in the distant past (Table 2), *Metulj-2_Hm* appears to be the youngest, with an average divergence from the consensus of ~5%. The topology of the *Metulj* tree generated as part of a COSEG [12] analysis supports the divergence analyses (Figure 2). For example, *Metulj-2_Hm* is a near-terminal node and exhibits the lowest level of divergence, while *Metulj-0_Hm* and *7*, which are estimated to be older are found nearer the root. Analyses of nested insertions via TinT [14] also supports this arrangement with *Metulj-0* and *7*, both of which exhibit high divergence levels, harboring proportionally more nested insertions than other subfamilies (Additional file 1: Figure S1). There does not appear to be any recent SINE activity in the *H. melpomene* genome. This could be due to inactivation and subsequent removal (see below) of the autonomous LINE partner for *Metulj*. Indeed, we are unable to identify the likely autonomous partner for this SINE family, because most older LINE families are present only as incomplete ‘fossils’ in the genome.

**Table 2:** **Divergence values and estimated activity periods for *Metulj *** **subfamilies**

**Metulj subfamily**	**Mean distance**	**Standard deviation**	**Range**	**Time (mya)**
Metulj-0_Hm	0.20747	0.06289	0.14458-0.27036	7.6-14.2
Metulj-1_Hm	0.17328	0.07409	0.09919-0.24737	5.2-13.0
Metulj-2_Hm	0.15970	0.09649	0.06321-0.25619	3.3-13.4
Metulj-5_Hm	0.20597	0.06798	0.13799-0.27395	7.2-14.4
Metulj-6_Hm	0.20272	0.07116	0.13156-0.27388	6.9-14.3
Metulj-7_Hm	0.24241	0.06665	0.17576-0.30906	9.2-16.2

Autonomous non-LTR elements exhibit a similar lack of recent activity with mean periods of activity ranging from ~2.7 mya to over 21 mya (Additional file 2: Table S1). A general lack of retrotransposition competence is suggested when examining numbers of potentially intact ORFs. We were unable to identify intact ORFs for most autonomous retrotransposon families and, of the families with identifiable, intact ORFs, the numbers were generally small. The largest number of intact ORFs was for RTE-3_Hm, with six (Table 3). The lack of success in identifying intact ORFs could be attributed to problems with the assembly. Most breaks in an assembly are associated with highly similar TE insertions. However, we were able to identify multiple instances of relatively long and highly similar sequences (see the discussion of Tc3-1_Hm below), suggesting instead that intact non-LTR ORFs, if present, would not evade detection.

**Table 3:** Counts of intact open reading frames for full length consensus sequences of each element class

**Class**	**Element name**	**Coordinates of ORF**	**# Intact**
DNA Transposon	**Tc3-1_Hm**	**120 - 1208**	**43**
NonLTR	Jockey-1_Hm	2980 - 4896	3
	Jockey-3_Hm	2051 - 4969	2
	L2-1_Hm	534 - 2924	1
	L2-7_Hm	95 - 1828	1
	L2-9_Hm	55 - 1530	3
	L2-13_Hm	543 - 2975	1
	L2-14_Hm	1468 - 4407	1
	L2-15_Hm	505 - 1986	1
	Proto2-3_Hm	111 - 1280	1
	R1-2_Hm	1411 - 4557	2
	R4-2_Hm	119 - 4207	1
	RTE-1_Hme	616 - 3636	1
	**RTE-3_Hm**	**264 - 3233**	**6**
	RTE-5_Hm	1334 - 3874	2
	RTE-9_Hm	723 - 1724	2
	RTE-10_Hm	323 - 1639	1
	RTE-15_Hm	69 - 1130	1
	RTE-20_Hm	181 - 3144	3
	TRAS1_R1_Hm	1299 - 3611	2
	Zenon-1_Hm	172 - 3333	1
	Zenon-2_Hm	590 - 3517	2
LTR	Gypsy-1_HMM-I	13 - 4542	1
	Gypsy-2_HMM-I	1071 - 5060	1
	Gypsy-3_HMM-I	2741 - 4402	1
	Gypsy-5_HMM-I	52 - 1716	1
	Gypsy-5_HMM-I	2601 - 4148	1
	Gypsy-6_HMM-I	84 - 2540	1
	**Gypsy-6_HMM-I**	**3008 - 4198**	**5**
	Gypsy-7_HMM-I	49 - 1272	1
	Gypsy-7_HMM-I	1694 - 3433	1
	Gypsy-8_HMM-I	1260 - 3167	1
	Gypsy-10_HMM-I	1525 - 3489	1

Counts were determined as describe in the text.

Bolded elements indicate the highest count in each category.

DNA transposons exhibit a much different pattern of succession with multiple lineages exhibiting relatively recent activity (i.e. mean activity periods estimated within the last 2 my; Additional file 3: Table S2). Only three autonomous DNA transposon families were identified in the genome but one stands out. Tc3-1_Hm exhibits an average divergence of 0.002% among 113 full length insertions. A total of 43 intact ORFs are present, suggesting that this family is a recent and active addition to the TE repertoire of *H. melpomene*. However, no intact transposase ORFs other than Tc3-1_Hm were evident. A second standout is the Helitron superfamily, which also appears to have undergone a relatively recent amplification and is the most prevalent Class II element, occupying ~5% of the genome. Several other element families also appear to be young and active. These include multiple nonautonomous families of the piggyBac, Mariner, hAT and Helitron superfamilies and the two autonomous piggyBac elements. For the purposes of this study, MITEs (miniature inverted repeat transposable elements) were considered a subset of non-autonomous DNA transposons.

### Evidence of TE removal

**Figure 3:**
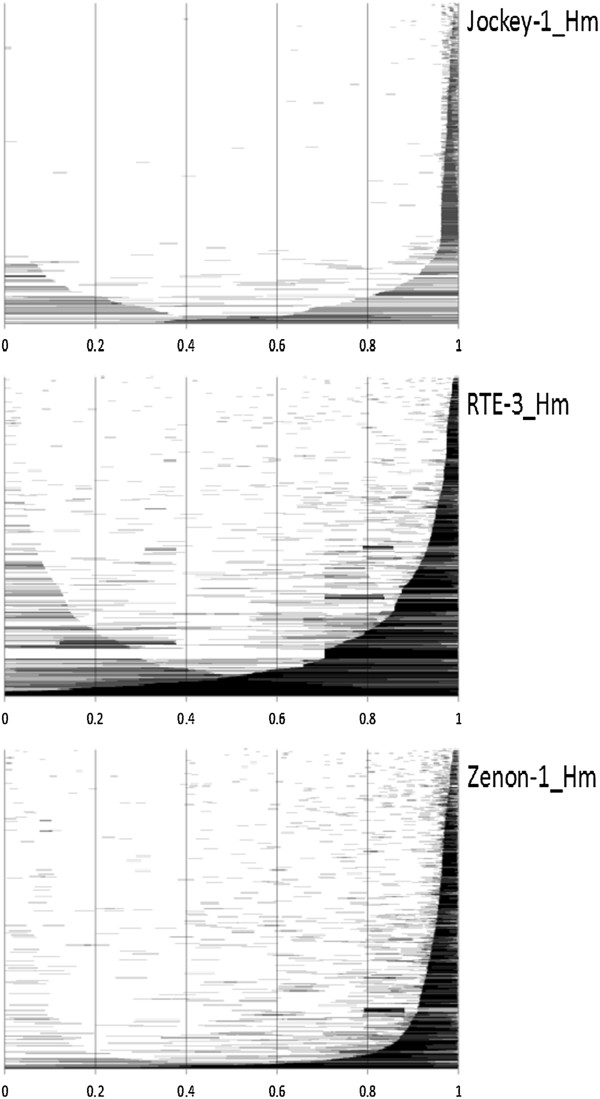
**Length distribution of three *H. melpomene *LINE insertions.** Insertions are ordered from bottom to top by length (longest insertions at the bottom). Numbers along the x-axis are normalized to reflect length proportions relative to the total length of the family consensus.

As part of their mobilization non-LTR retrotransposons are reverse transcribed from their 3′ end. Large non-LTR retrotransposons are often truncated at the 5′ end and this is thought to be a consequence of either premature dissociation of reverse transcriptase or the activity of cellular RNases [15]. However, the presence of a 5′ region without the corresponding 3′ region is not likely to be result of either process. Thus, LINE fragments that lack their 3′ ends or consist solely of internal sections are considered evidence of genomic deletions as described previously [16,17]. We found that many *H. melpomene* LINE families exhibited patterns consistent with large deletions acting to remove them from the genome (Figure 3 and Additional file 4: Figure S2). As expected given their insertion mechanism, we observe an abundance of 3′ fragments for LINE families. However, unlike what is observed in mammals [16], which exhibit a low rate of DNA loss, we see a large number of 5′ fragments and orphaned internal LINE fragments. This suggests ectopic recombination acting to remove these elements from the genome at a high rate.

### Evolutionary relationships among autonomous Non-LTR retrotransposons

**Figure 4:**
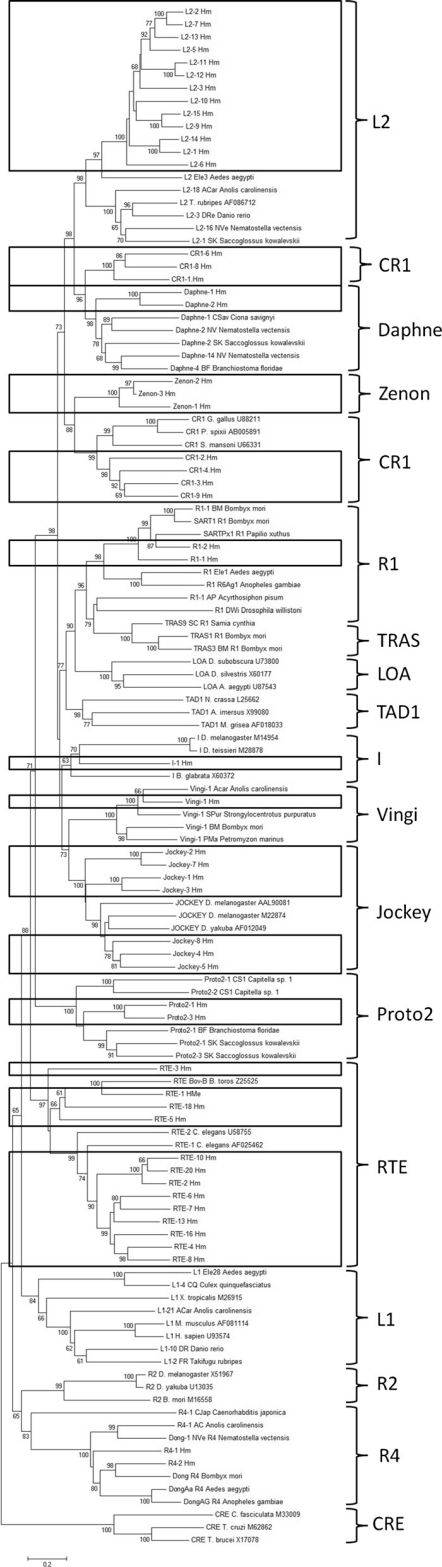
**Phylogenetic relationships of autonomous non-LTR elements.** Relatively weak bootstrap values (< 65) were not included.

A maximum-likelihood tree of autonomous non-LTR retrotransposons (Figure 4) reveals that the *H. melpomene* genome harbors 56 families from 10 diverse clades (L2, CR1, Vingi, Daphne, R1, I, Jockey, Proto2, RTE and R4). Although most clades (7/10) have relatively low diversity (three or fewer representatives within the clade), the remaining clades are represented by many families. The L2 and RTE clades are each represented by 13 families, while the Jockey and CR1 clades each contain seven. Zenon is sometimes considered a member of the CR1 clade, thereby raising the count to ten for that family. Although most of the non-LTR consensus sequences that were generated cluster with their appropriate clade, three CR1 families (CR1-6_Hm, CR1-8_Hm, CR1-1_Hm) fail to do so with bootstrap support (greater than 65). Despite the fact that RepeatMasker identifies these elements as CR1, these families form a monophyletic group sister to Daphne elements and may represent a novel clade.

### Horizontal transfer

**Figure 5:**
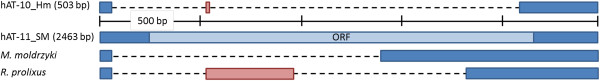
**Relationships among hits highly similar to hAT-10_Hm in other taxa.** Comparisons are to hAT-11_SM, the consensus sequence of a known autonomous DNA transposon from the planarian, *Schmidtea mediterranea*, and contigs from *M. moldrzyki* and *R. prolixus*. Blue boxes exhibit high similarity within the corresponding regions. Red boxes found for *H. melpomene* (nine bases) and *R. prolixus* (410 bases) indicate regions with no similarity to any corresponding sequence in the other taxa. Contig IDs and sequence similarity values are available from the text.

We considered an element to be a likely candidate for horizontal transfer (HT) if a BLASTN search indicated that the consensus shared >95% sequence identity over at least 80% of its length. BLAST results from querying NCBI’s WGS database suggest three candidate elements for horizontal transfer between *H. melpomene* and other animals (Figure 5). The first involves a non-autonomous hAT-like element, *nhAT-10_Hm* with hits to scaffolds in *Rhodnius prolixus* (best hit = 97% identity over 83% of the query, E-value = 0)*, Mengenilla moldrzyki* (96% identity over 83% of the query, E-value = 0)*,* and *Schmidtea mediterranea* (95% identity over 83% of the query, E-value = 0)*. R. prolixus* and *M. moldrzyki* are insects from the orders Hemiptera and Strepsiptera, respectively. The fact that similar hits were not observed in more closely related taxa such as *B. mori* or *D. plexipus* is evidence that these elements were likely transferred to the genome by mechanisms other than vertical transmission.

The other two candidates were piggyBac-1_Hm and piggyBac-2_Hm with hits matching our criteria in *Manduca sexta* (piggyBac-1_Hm, 99% identity over 100% of the query, E-value = 0), *Bombyx mori* (and piggyBac-2_Hm, 99% identity over 100% of the query, E-value = 0), and *D. plexipus* (and piggyBac-2_Hm, 98% identity over 85% of the query, E-value = 0). In the case of *D. plexipus*, the reduced coverage is due to the fact that the insertion terminates with the scaffold (AGBW01001888).

## Discussion

### *TE content in* Heliconius *compared to* Bombyx

The genome of *Heliconius melpomene* is the third lepidopteran genome to be fully sequenced. Unfortunately, the authors of the monarch genome manuscript did not complete a comprehensive analysis of the TE landscape [10], and our comparisons were therefore limited to *B. mori*.

TEs make up 35% of the *B. mori* genome, with the largest fraction (26.6%) being non-LTR retrotransposons [5]. Of the non-LTR content, around half is derived from SINEs, 48%. A smaller fraction, ~25%, of the *H. melpomene* genome is composed of TEs. 12.5% consists of non-LTR retrotransposons, and 8% of the genome is occupied by SINEs (68% of the non-LTR content). Thirty-two non-LTR families belonging to 12 clades (Jockey, RTE, CR1, CRE, R1, R2, R4, I, Vingi, Daphne, Proto2 and L2) were identified and classified from *B. mori*. This is two more than were identified in *H. melpomene*. However, despite harboring two fewer clades than *B. mori*, the *H. melpomene* genome contains more families in total and this can be attributed to higher within-family diversity in some clades. For instance, 13 families of L2 and 10 families of CR1 were identified in *H. melpomene,* while only one and two are present in *B. mori*, respectively. Of the available lepidopteran genomes (including the monarch butterfly), *Metulj* is restricted to *Heliconius*.

In *H. melpomene*, LTR retrotransposons make up only ~0.45% of the genome. This is within the same range as what was described for *B. mori* by Osanai-Futahashi et al. in 2008 [5], 1.7%, but substantially different from a second estimate of LTR content in *B. mori* by Jin-Shan et al. [18], 11.8%. Given that Osanai-Futahashi examined a more complete assembly of the silkworm genome, we suspect that their estimate is closer to reality. Both genomes harbor Gypsy and Copia elements. *B. mori* however has two additional families which include Pao and Micropia [5]. That being said, ~2.4% of the genome consists of candidate TEs that remain unidentified by our analyses and could belong in the LTR category.

While the retrotransposon content of *B. mori* and *H. melpomene* are similar, with regard to Class II elements, the DNA transposons, the two species are strikingly different in both content and quantity. Only ~3% of the *B. mori* genome consists of Class II elements [5] while ~10% of the *H. melpomene* genome is derived from DNA transposons. Indeed, the butterfly genome has been the subject of considerable DNA transposon activity within the recent past. This includes massive amplification by the Helitron superfamily and very recent, if not ongoing activity, from one member of the Tc-Mariner family. At least 43 intact members of the Tc3-1_Hm autonomous element are present in the genome draft and they are 99.4% identical, indicating that these elements are likely active.

### *Turnover of non-LTR element families in* Heliconius

The lack of intact, older LINE elements in the genome suggests that they have a high fitness cost and that they may be preferentially removed. Mechanisms to accomplish removal include ectopic recombination between similar elements and removal of individual insertions via selection. Indeed, increased rates of ectopic recombination have been suggested as a mechanism for the differences in TE accumulation in both mammals and insects [19]. Our results suggest that this mechanism is in play in the *H. melpomene* genome. Figure 3 indicates that deletions of large portions of LINE elements occur at relatively high frequency.

That being said, we note that other elements families have accumulated to relatively high numbers. In particular, this is true of *Metulj* and many of the Helitron elements. However, those elements with high copy numbers are typically under 500 nt in length. Previous authors have noted that shorter elements are likely less prone to recombination than their longer cousins [20,21], allowing them to remain in the genome.

Hierarchical insertion patterns (TinT) indicate short periods of activity for the longer, autonomous elements, which exhibit a clear pattern of succession (Additional file 1: Figure S1). If one ignores the wide distributions of *Metulj*, the only SINE, each non-LTR family occupies a relatively narrow temporal space indicating that they experience brief periods of activity before ceasing mobilization. This is similar to what has been observed in some other taxa, including the lizard *Anolis carolinensis*, but is distinct from mammals, which have a single lineage of LINE-1 that has accumulated high copy numbers [22]. The same analysis was performed for *B. mori*, with similar results (Additional file 1: Figure S1). Like many insects, the *H. melpomene* genome is relatively small, ~269 Mb. These results suggest that, while TE activity occurs and novel elements can invade the genome with some success, strong selection is working against the accumulation of large TEs and that homologous recombination acts to rapidly disable elements and keep the genome compact.

### Evidence of horizontal transfer

We found evidence of horizontal transfer of three DNA transposons between *H. melpomene* and other taxa. Multiple elements matching nhAT10_Hm were identified in three taxa, the triatomine bug, *Rhodnius prolixus*, a strepsipteran insect, *Mengenilla moldrzyki*, and the planarian, *Schmidtea mediterranea*. In each case, the entire nhAT10_Hm is present as part of a larger element. For example, when compared to the planarian autonomous element, hAT-11_SM, nhAT10_Hm_has the hallmarks of an internal deletion variant. The first 70 bases are essentially identical between both TEs, as are the last 420 (Figure 5). The same regions overlap with as yet unnamed repeats in *R. prolixus* and *M. moldrzyki*. The top hit for *R. prolixus* can be found on contig ACPB02011601.1, nt 29253–30319, and the top hits for *M. moldrzyki* can be found on contigs AGDA01050831.1, nt 10068–10485 and AGDA01007612.1, nt 6860–6920, respectively. In these two taxa, the overlaps are with elements that are likely nonautonomous. This suggests that a hAT-22_SM-like element has been invading multiple genomes and produced similar nonautonomous variants in each. Indeed, we subsequently used BLASTN to query the genome drafts of *H. melpomene*, *M. moldrzyki* and *R. prolixus* using the consensus sequence of hAT-11_SM and, while no full-length elements were obvious, we identified high scoring (E-value = 0) hits from various portions of the consensus in each. Interestingly, both *S. mediterranea* and *R. prolixus* have been implicated in horizontal transfer previously [23-25].

The other candidates are the autonomous piggyBac elements, piggyBac-1_Hm and piggyBac-2_Hm. A single instance of *piggyBac-1_Hm* was identified in the *Manduca sexta* genome draft (scaffold AIXA01012877) with 99% identity over its entire length. Two full length copies of *piggyBac-2_Hm* in the Dazao strain of *B. mori* (scaffolds AADK01008943 and AADK01013248) with the same values. The final hit, to the monarch butterfly genome, is incomplete due to the termination of the scaffold ~350 bp prior to the end of the consensus. Both moths would have diverged from the lineage leading to butterflies ~145 mya [26] while the monarch is thought to have diverged from *Heliconius* ~89.79 mya [27] and, given the high rate of change observed in lepidopteran genomes, it is unlikely that they would have been conserved over such an extended period. This suggests to us that horizontal transfer explains their presence in each. However, as additional genomes are characterized this interpretation could change.

## Conclusions

In conclusion, by conducting the first full TE analysis of a butterfly we have demonstrated that TEs, specifically SINEs and Helitrons, make up a large portion of the *H. melpomene* genome. We identified a novel SINE family which is found only in *Heliconius* and demonstrated that the genome of *H. melpomene* has experienced recent DNA transposon activity, most notably a Tc3 element. We have also shown that older, intact LINE elements are not found within the genome and that their activity period in the genome is short due to their rapid removal. Further studies of other lepidopteran genomes will be beneficial to our understanding of TEs in lepidopterans.

## Methods

The genome sequence of a male *Heliconius melpomene melpomene* was recently described [8]. Briefly, the specimen was acquired from Darien, Panama and the genome was sequenced using both 454 and Illumina platforms to generate a 38X draft genome. The sequenced male was inbred for five generations of sib mating. Repeat discovery was performed as summarized elsewhere [8] and described briefly here. Repetitive sequences in the *H. melpomene* draft sequence (Genbank accession number: CAEZ01000000) were identified *de novo* using RepeatModeler [28]. To infer the consensus sequences for each repeat, we used the filtered RepeatModeler output to query the entire WGS draft using BLAST v2.2.23 [29]. Up to fifty of the top hits spanning at least 100 bases were extracted along with up to 1,000 bases of flanking sequence, and we aligned the extracted sequences with MUSCLE 4.0 [30] to generate 50% majority rule consensus sequences. Consensus sequences were considered ‘complete’ when single copy sequence could be identified at the 5′ and 3′ ends in each component sequence. If this condition was not met, the process was repeated until single copy DNA sequence was identifiable at both ends. The resulting library was submitted to CENSOR [31], BLASTN and BLASTX to ascertain the identity of the consensus with regard to previously classified elements. The result was a custom library of elements, which served as our library for subsequent analyses. The library of TEs was passed through a locally implemented version of RepeatMasker [32] to estimate the TE content of the *H. melpomene* genome.

### Identification of SINE subfamilies

We identified 14,196 intact insertions of *Metulj* between 240–294 bases in length (+/− 10% of the general consensus) and passed them to COSEG [12,13] for subfamily identification. COSEG examines multiple instances of TE insertions and identifies significant co-segregating (2–3 bp) sites in an effort to determine subfamily structure. A perl script provided by R. Hubley was used to refine the consensus sequence for each subfamily and is available upon request. We created a custom RepeatMasker library consisting of the suggested *Metulj* subfamily consensus sequences and extracted the top 150 hits for each from the genome. We aligned the extracted sequences with their respective subfamily consensus sequence to confirm the presence of each in the genome.

### Identification of intact ORFs

We submitted the consensus sequence of each TE to NCBI ORF finder to identify potential open reading frames (ORFs). We classified any elements with identifiable ORFs spanning 1000 bp or more as potentially full length. ORF sequences were translated and BLASTP was used to confirm identity. ORFs of BEL-1_HMM, BEL-2_HMM, Copia-1_HMM, Gypsy-10_HMM, Gypsy-1_HMM, Gypsy-2_HMM, Gypsy-3_HMM, Gypsy-4_HMM, Gypsy-5_HMM, Gypsy-6_HMM, Gypsy-7_HMM, Gypsy-8_HMM, Gypsy-9_HMM as well as RTE-1_HMe, R4-1_Hme were identified by other parties and were obtained from RepBase.

We estimated the number of intact ORFs for each family of autonomous elements by passing the ORF sequences through a local version of TBLASTN, after which, up to 50 of the top hits based on bit score were extracted with 1000 bp of buffer and aligned. Extracted sequences were trimmed so they began and ended at the same position as the ORF query sequence. We defined an intact ORF as one that is greater than or equal to 90% of the expected amino acid length, contains a single, terminal stop codon, and begins with a methionine start codon.

### Age analyses and relative insertion periods

We used the TinT online server (http://www.compgen.uni-muenster.de) as a method to determine periods of relative TE activity and succession patterns [14]. Due to low copy numbers, analysis of LTR elements could not be performed. Furthermore, DNA transposons utilize a cut-and-paste mechanism of transposition that makes a nested insertion analysis of this type less informative. Thus, we analyzed only non-LTR retrotransposons.

We also estimated activity periods based on genetic distances between individual insertions and the consensus of each subfamily as described previously [33,34]. Briefly, we created a modified TE library consisting of the full consensus of all *Metulj* subfamilies and non-autonomous DNA transposons, the full ORFs of all DNA transposons and 500 bp from the 3′ end of non-LTR ORFs. This library was then used to query the genome using RepeatMasker. We estimated Kimura2-parameter [35] distances (including CpG sites) between each insertion and its respective consensus [33]. A neutral mutation rate is not available for *H. melpomene.* We applied an estimated mutation rate of 0.01909 substitutions per site/per million years which was taken from Papilioninae, a subfamily of the butterfly family Papilionidae [36].

The nearly vertical succession of non-LTR retrotransposons seen in the TinT plot (Additional file 1: Figure S1) suggests a rapid turnover of longer elements. One mechanism through which elements can be removed from a genome is non-homologous recombination leading to large deletions. By taking each RepeatMasker hit from each TE subfamily and mapping its location along the consensus element, we were able to examine decay patterns among selected elements.

### Evolutionary relationships among autonomous non-LTR retrotransposons

From Genbank and Repbase, we collected non-LTR retrotransposon protein sequences from diverse known clades [37,38]. We aligned these sequences with the consensus sequences retrieved from the *H. melpomene* genome using Clustal W in BioEdit [39]. The most conserved region (about 300 amino acids) from the reverse transcriptase domain was identified and used in the phylogenetic analysis. Newly identified families missing this region were excluded. We inferred a maximum-likelihood tree with 1,000 bootstrap replicates using MEGA5 [40].

### Horizontal transfer

We investigated the taxonomic distribution of all *H. melpomene* TEs by querying the full WGS database at NCBI with BLAST. We considered an element to be a likely candidate for HT if a BLASTN search indicated that the consensus shared >95% sequence identity over at least 80% of its length. Any hits matching these criteria were examined by extracting the highest scoring hits, alignment to the query sequence and manual examination.

## Abbreviations

TE: Transposable element; Non-LTR: Non-long terminal repeat; LTR: Long terminal repeat; LINEs: Long INterspersed Elements; SINEs: Short INterspersed Elements; ORF: Open reading frame; TIR: Terminal inverted repeats; TinT: Transposition in Transposition.

## Competing interests

The authors declare that they have no competing interests.

## Authors’ contributions

CAL and DAR conducted analyses and participated in writing the manuscript. RNP provided perl scripts for analysis and participated in conducting the analysis of TE removal. PAN participated in writing the manuscript and conducting phylogenetic analysis. BAC contributed analyses and participated in writing the manuscript. All authors read and approved the final manuscript.

## Supplementary Material

Additional file 1: Figure S1 Results of theTinT analysis for *H.melpomene* (A) and *B. mori* (B) non-LTR elements. TinT uses patterns of nested insertion to predict relative activity periods among TEs. In the graph, periods of probable activity are depicted by an oval (period of maximum activity), vertical lines (95% of the probable activity period), and horizontal lines (99% of the probabl activity period). Details are available in [14]. Click here for file

Additional file 2: Table S1 Estimated ages of Non-LTRs. Ages were calculated as described in the text for Table 2. Click here for file

Additional file 3: Table S2 Estimated ages of DNA transposons. Ages were calculated as described in the text for Table 2. Click here for file

Additional file 4: Figure S2 Length distributions of *H. melpomene* LINE insertions. Details are as described in Figure 3. Click here for file
