# Supplementary material for: Transposable element evolution in Heliconius suggests genome diversity within Lepidoptera
Source: Mob DNA. 2013 Oct 2;4:21. doi: 10.1186/1759-8753-4-21 (PMC4016481; doi:10.1186/1759-8753-4-21)

Supplemental Figure S1: Results of theTinT analysis for *H.melpomene* (A) and *B. mori* (B) non-LTR elements. TinT uses patterns of nested insertion to predict relative activity periods among TEs. In the graph, periods of probable activity are depicted by an oval (period of maximum activity), vertical lines (95% of the probable activity period), and horizontal lines (99% of the probabl activity period). Details are available in [14].

A.

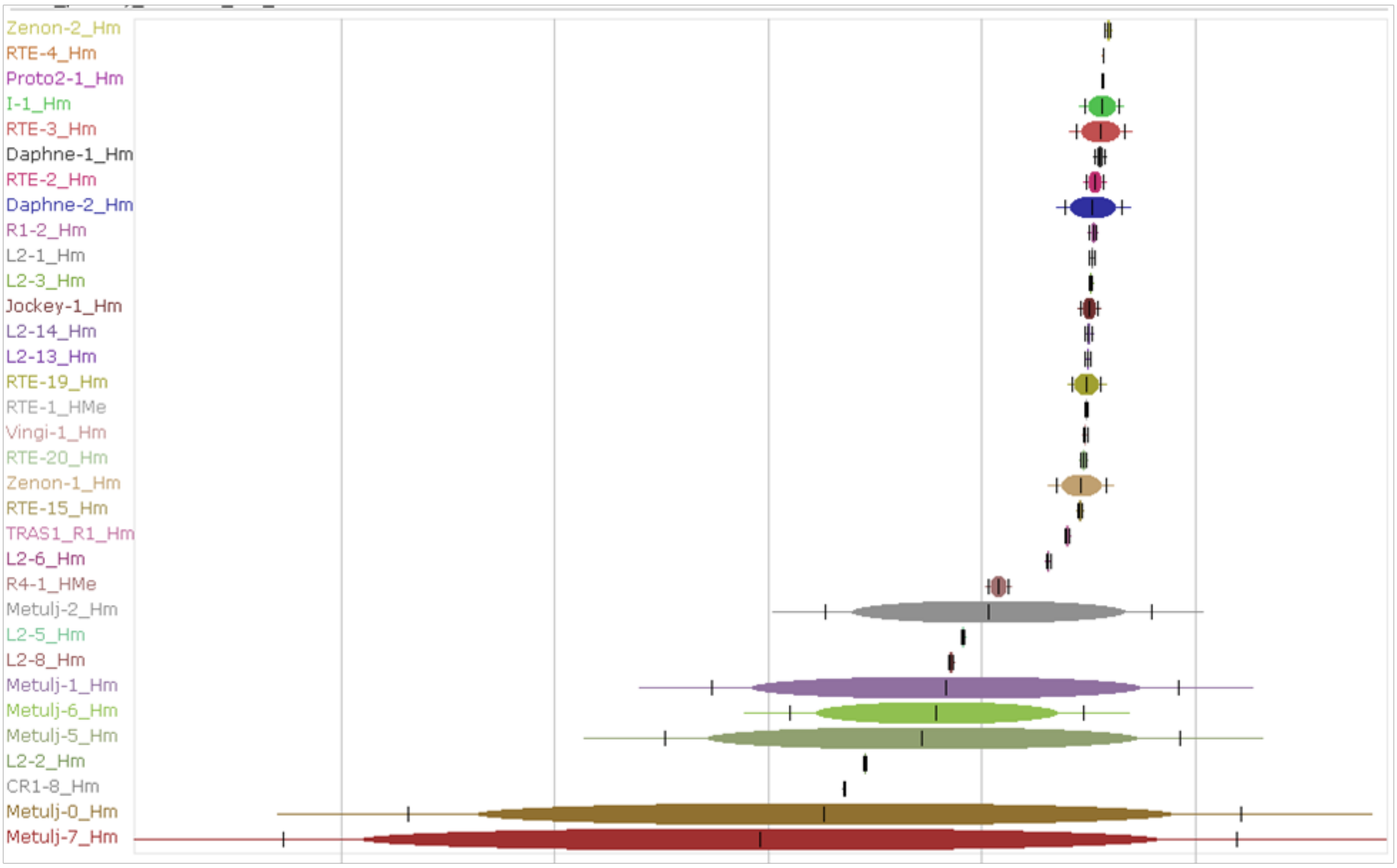

B.

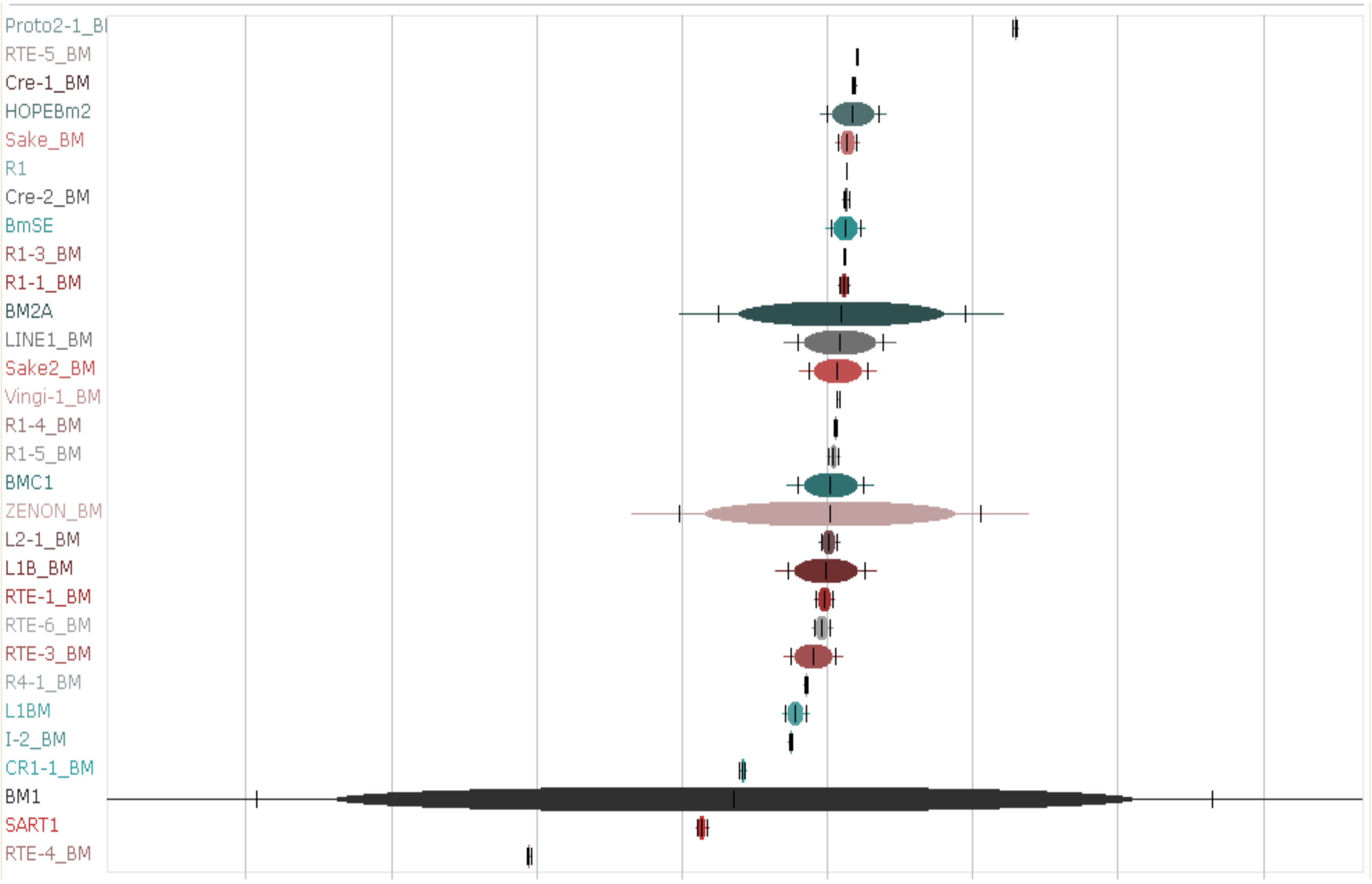

Supplement: Additional file 1: Figure S1 — Results of theTinT analysis for H.melpomene (A) and B. mori (B) non-LTR elements. TinT uses patterns of nested insertion to predict relative activity periods among TEs. In the graph, periods of probable activity are depicted by an oval (period of maximum activity), vertical lines (95% of the probable activity period), and horizontal lines (99% of the probabl activity period). Details are available in [14]. [file 1759-8753-4-21-S1.pdf]
