# Supplementary material for: Transposable element evolution in Heliconius suggests genome diversity within Lepidoptera
Source: Mob DNA. 2013 Oct 2;4:21. doi: 10.1186/1759-8753-4-21 (PMC4016481; doi:10.1186/1759-8753-4-21)
Supplement: Additional file 4: Figure S2 — Length distributions of H. melpomene LINE insertions. Details are as described in Figure 3. [file 1759-8753-4-21-S4.pdf]

Figure S2: Length distributions of *H. melpomene* LINE insertions. Details are as described in Fig. 3.

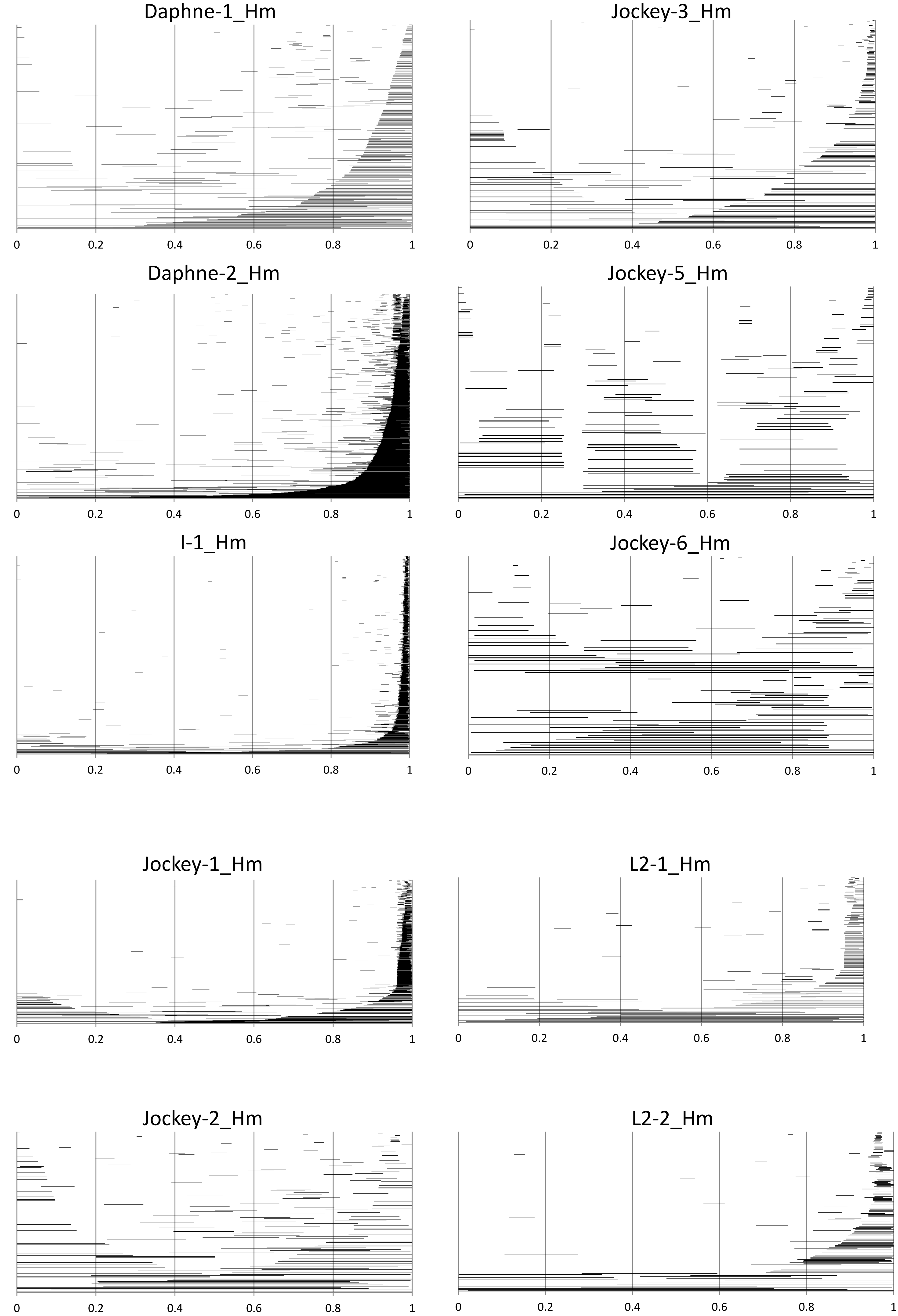

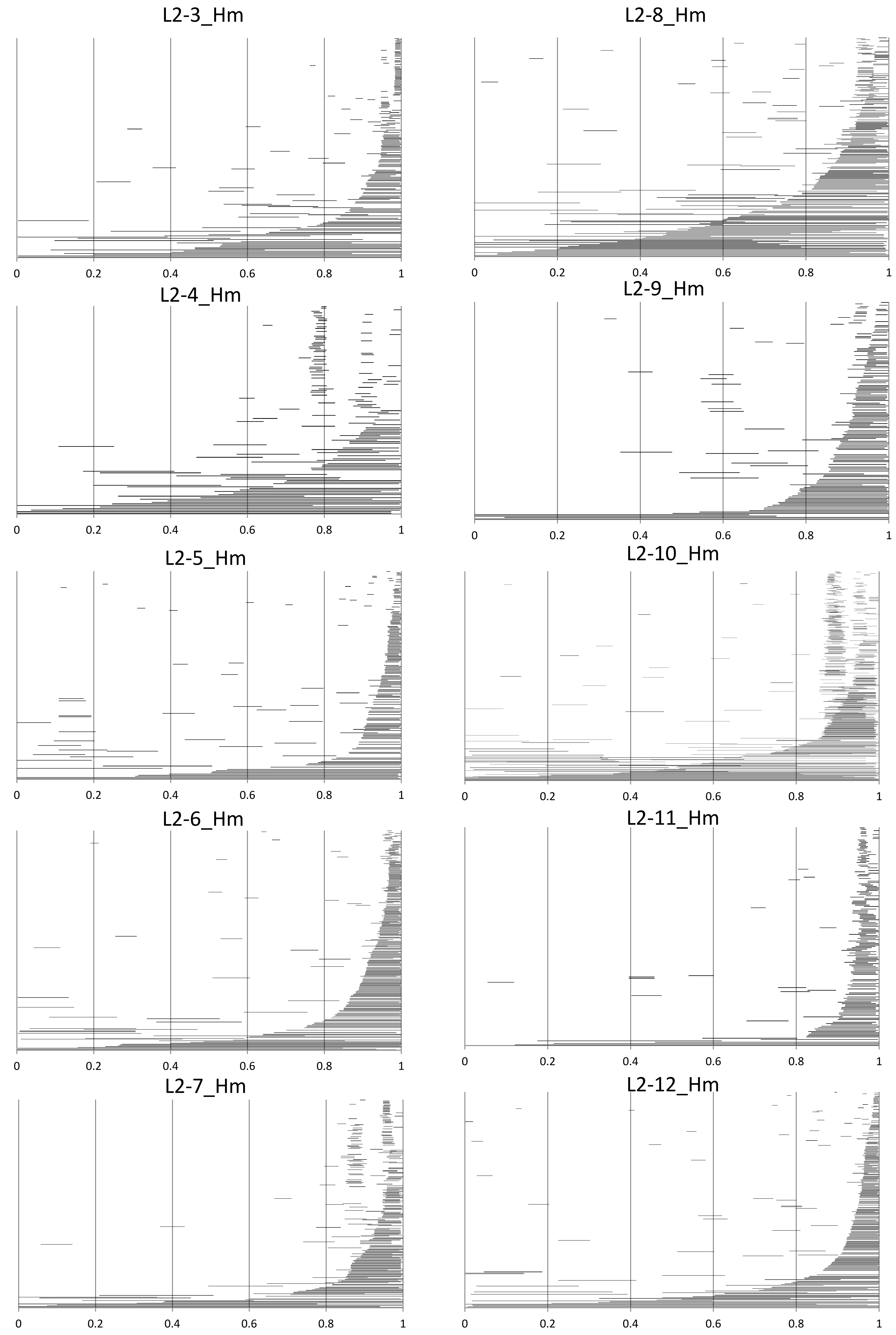
L2-3_Hm
L2-8_Hm
L2-4_Hm
L2-9_Hm
L2-5_Hm
L2-10_Hm
L2-6_Hm
L2-11_Hm
L2-7_Hm
L2-12_Hm
0
0.2
0.4
0.6
0.8
1

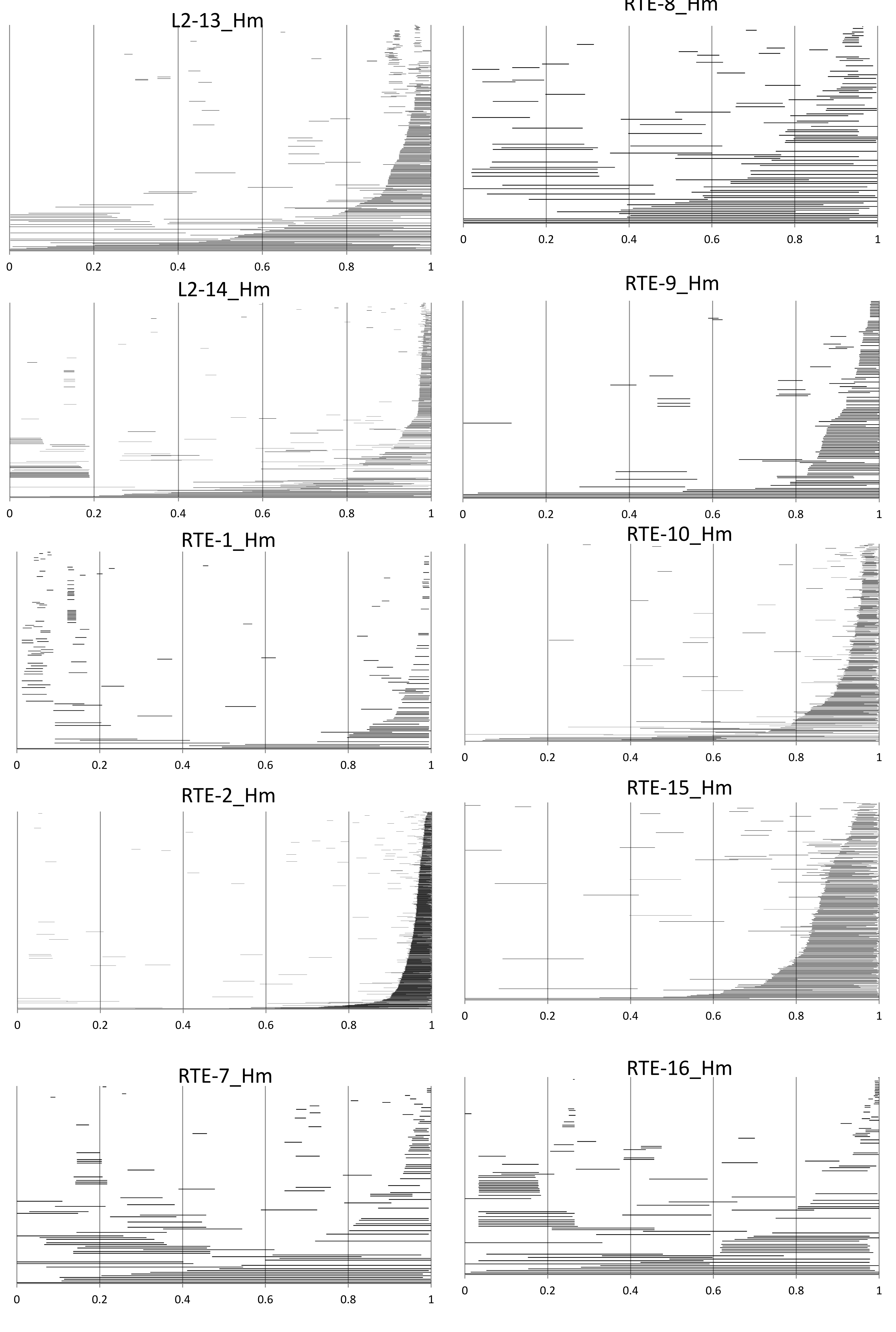
L2-13_Hm
RTE-8_Hm
L2-14_Hm
RTE-9_Hm
RTE-1_Hm
RTE-10_Hm
RTE-2_Hm
RTE-15_Hm
RTE-7_Hm
RTE-16_Hm
0
0.2
0.4
0.6
0.8
1

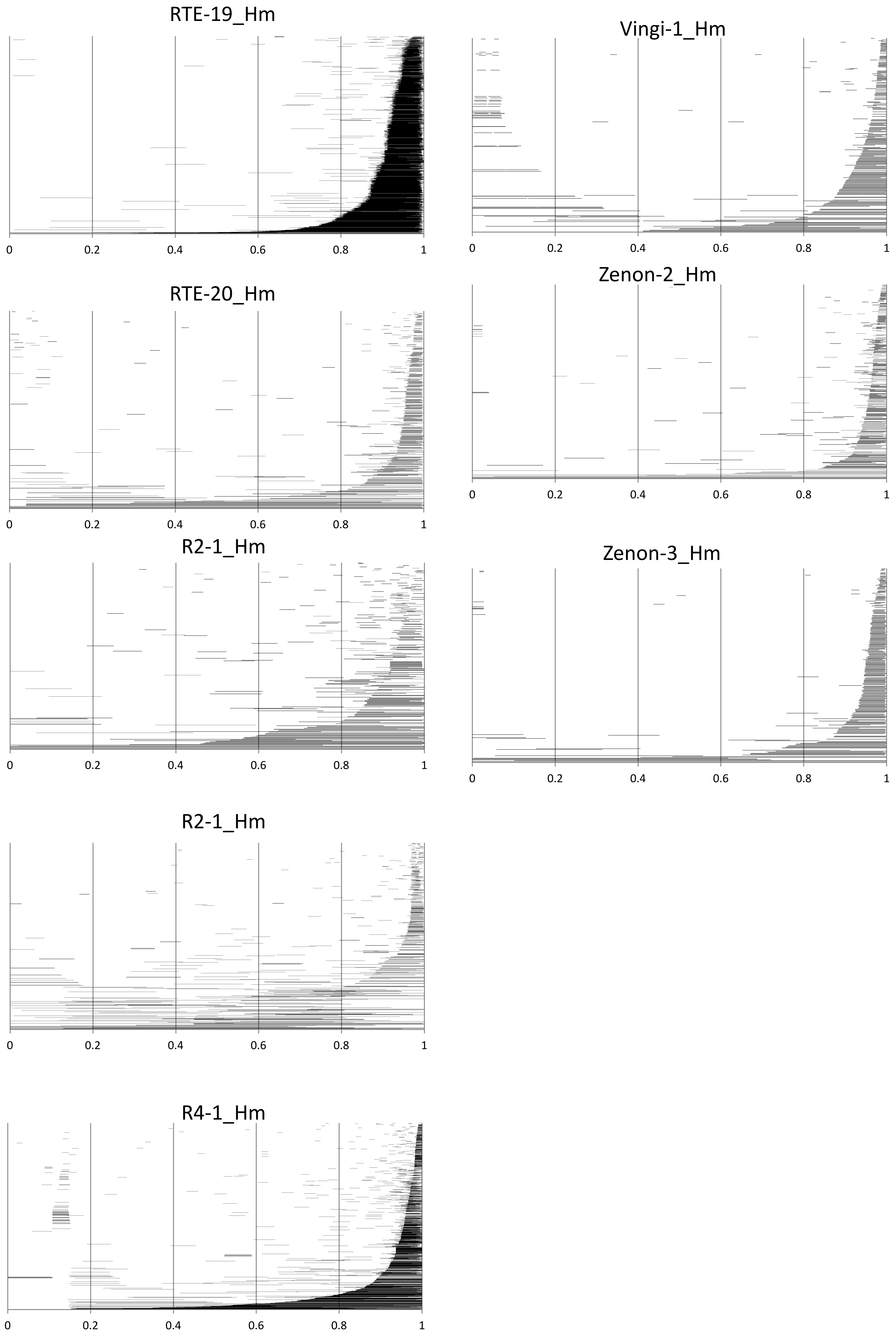
RTE-19_Hm
Vingi-1_Hm
RTE-20_Hm
Zenon-2_Hm
R2-1_Hm
Zenon-3_Hm
R2-1_Hm
R4-1_Hm
0
0.2
0.4
0.6
0.8
1
